# Development of a Chatbot Program for Follow-Up Management of Workers’ General Health Examinations in Korea: A Pilot Study

**DOI:** 10.3390/ijerph18042170

**Published:** 2021-02-23

**Authors:** Byeong Jin Ye, Ju Young Kim, Chunhui Suh, Seong Pil Choi, Maro Choi, Dong Hyun Kim, Byung Chul Son

**Affiliations:** 1 Department of Occupational and Environmental Medicine & Institute of Environmental and Occupational Medicine, Busan Paik Hospital, Inje University, Busan 47392, Korea; yebj_oem@paik.ac.kr (B.J.Y.); chsuh@paik.ac.kr (C.S.); oemos83@gmail.com (S.P.C.); akfh1325@gmail.com (M.C.); oemkdh@gmail.com (D.H.K.); 2 Colorbot Inc., Busan 49324, Korea; jooykim@pusan.ac.kr

**Keywords:** chatbot, follow-up management, occupational health service, workers’ general health examination

## Abstract

(1) Background: Follow-up management of workers’ general health examination (WGHE) is important, but it is not currently well done. Chatbot, a type of digital healthcare tool, is used in various medical fields but has never been developed for follow-up management of WGHE in Korea. (2) Methods: The database containing results and explanations related to WGHE was constructed. Then, the channel, which connects users with the database was created. A user survey regarding effectiveness was administered to 23 healthcare providers. Additionally, interviews on applicability for occupational health services were conducted with six nurses in the agency of occupational health management. (3) Results: Chatbot was implemented on a small scale on the Amazon cloud service (AWS) EC2 using KaKaoTalk and Web Chat as user channels. Regarding the effectiveness, 21 (91.30%) rated the need for chatbots as very high; however, 11 (47.83%) rated the usability as not high. Of the 23 participants, 14 (60.87%) expressed overall satisfaction. Nurses appreciated the chatbot program as a method for resolving accessibility and as an aid for explaining examination results and follow-up management. (4) Conclusions: The effectiveness of WGHE and the applicability in the occupational health service of the chatbot program for follow-up management can be confirmed.

## 1. Introduction

A health examination provides an effective screening and medical evaluation strategy that has been scientifically shown to facilitate early detection and treatment, even among asymptomatic patients, thereby preventing disease and promoting personal health [1]. In Korea, business owners are obligated by the Industrial Safety and Health Act to participate in the workers’ general health examination (WGHE) program as one element of efforts to protect workers’ health. The general health examination covered by the National Health Insurance Service can be substituted for the WGHE [2]. Since 2009, the general health examination in Korea has identified cardiocerebrovascular disease as the main target disease, and the program has focused on improving lifestyles and strengthening follow-up management through a health risk assessment [3]. 

Follow-up management for the general health examination entails additional interventions, such as diagnosis confirmation, education, and counselling, regarding results of the examination for those who need further action after the screening [4]. The importance and effectiveness of follow-up management are well known from previous studies. In many studies, the risk factors for cardiocerebrovascular diseases, such as smoking, drinking, exercise, eating habits, blood pressure, and blood sugar, were improved in the group with specific follow-up management, compared to the group without it [5,6,7]. However, in the past studies in Korea, only 35.5% of people who needed a follow-up confirmation examination after the initial examination followed through with the confirmation examination [8]. Also, only 2.21% and 1.18%, respectively, of patients who needed hypertension and diabetes treatment based on the general health examination were treated within 90 days [2]. These results indicate that even if a medical issue is identified during the general health examination, proper follow-up is often neglected.

Chatbot, a type of digital healthcare tool, is a rule-based or artificial intelligence-based communication software that uses a mobile device to provide answers and relevant information in response to questions posed through text or voice conversations [9]. This technology is increasingly used for applications in credit scoring [10] and marketing strategies [11] due to the universalization of smart devices and mobile (online) communication and the expanding influence of messenger apps. Recently, chatbots have been increasingly used as a tool for digital healthcare. For example, the chatbot program “Kohby” at Kangbuk Samsung Hospital provides information on health check-ups and administrative services such as appointments or payments [12]. In addition, the chatbot also provides appropriate answers to questions about symptoms and diseases, and connects the patient with an appropriate doctor through “HealthTap” [13] and “Babylon Healthcare” [14]. The chatbot additionally provides information regarding treatment and management for cancer patients [15,16] or interventions for stress [17] or mental health problems [18]. Furthermore, chatbot also plays a role in motivating and sustaining lifestyle changes, for example, quitting smoking [19,20]. 

Because chatbots are optimised for mobile devices and can therefore obtain the necessary information without the need to install a separate app, the number of users and the service area are becoming increasing significantly. However, no chatbot program has yet been developed that explains the results of the general health examination explicitly and provides methods for follow-up management, that are easily understood by the general public. 

This pilot study is the first step in a plan to develop a chatbot program for follow-up management of the WGHE. The study develops an early version of a chatbot program that generates health status information and health management methods based on individual results from the WGHE. Furthermore, this study investigates the effectiveness of the chatbot program to healthcare providers who have undergone WGHE program in 2018 and the applicability of the chatbot program to nurses in the special agency of occupational health management in charge follow-up management of WGHE.

## 2. Materials and Methods 

### 2.1. Setting and Participants

Research participants were recruited from October to November 2019. The research director visited three hospitals and explained the purpose of the study, the contents of the study, and the method of participation for those who received the WGHE in 2018. In addition, consent was obtained from those who agreed to participate in the study. Subsequently, chatbot authentication and personal data were collected after using the chatbot program for a week, a survey was conducted online. Research participants submit their own phone number, and the researcher sends a channel subscription message to the participant’s phone number. It is authenticated through the consent of the channel subscription, and data is generated based on the inputted health examination results and information appropriate to one’s health status is sent to the individual. Also, the chatbot program was explained to nurses working at the agency of occupational health management. They checked the entire contents of the chatbot program for a week and then participated in the interview. The study was approved by the Institutional Review Board of Busan Paik Hospital, Inje University (No. 19-0189). 

### 2.2. Chatbot Program Development

The chatbot program was developed based on the rule-based or scenario method that is most often used to describe professional content. The development process is as follows.

1. Requirements analysis: Define business requirements for program development.
2. Scenario flow definition: Define the flow of core tasks to which the program is applied and the flow design for providing examination results.
3. Database construction: Design and build the schema for the examination results and additional explanation database. At this time, construct images and videos that will be useful for explaining the examination results.
4. Channel establishment: Set the channel where the user and chatbot will meet.
5. Chatbot program development: Develop a content-provided chatbot program and a chatbot program that extracts and provides necessary answers.

**Figure 1:**
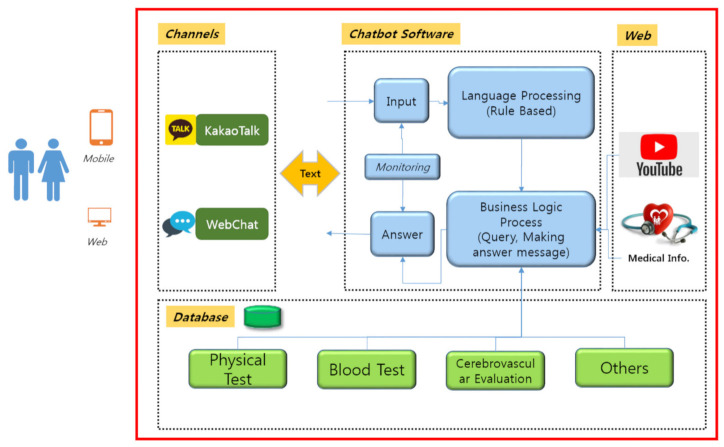
The overall system configuration of the chatbot program.

The chatbot program for follow-up management of the WGHE is implemented on a small instance of the Amazon cloud service (AWS) EC2 and web server; the development language node is 7.0, and the database management system is MySQL 5.7. KakaoTalk and Web Chat are used as user channels. After the user performs personal authentication through a smartphone, the program provides follow-up information based on individual results. Using the health examination results, a scenario-based process is used to create a response by linking to or searching the database, web search engine, and YouTube API (Application Programming Interface) for data suitable for the patient’s status and examination results (Figure 1). 

**Figure 2:**
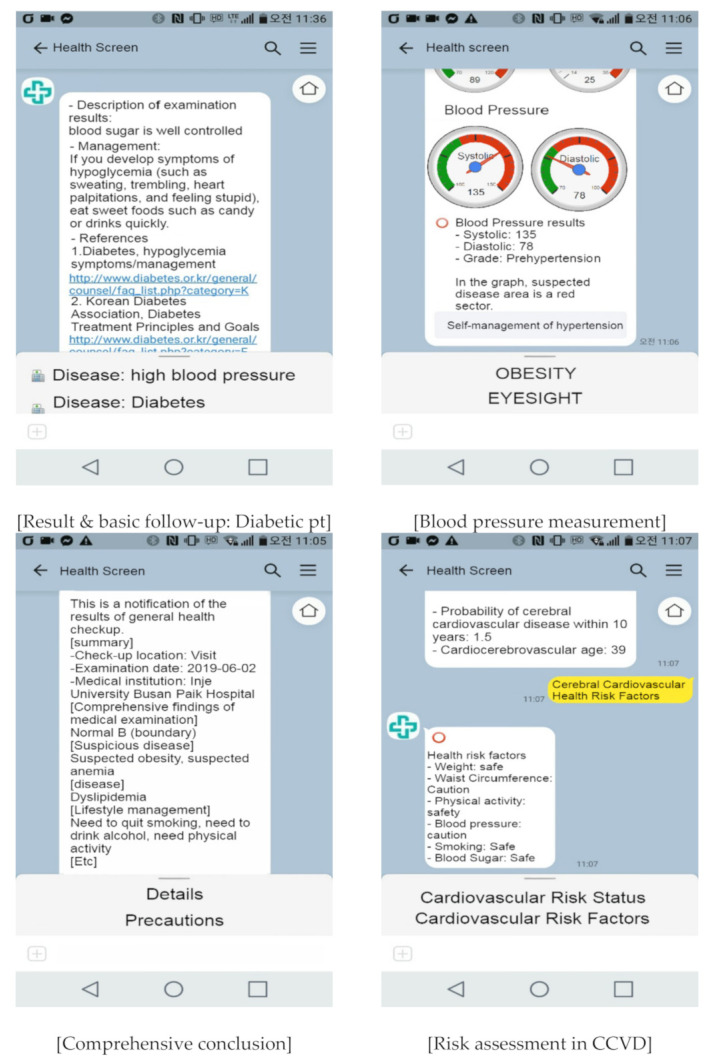
Contents of the chatbot program implemented on a smartphone.

Personal authentication is required only once, at the first access, and subsequent identification is automatic based on the phone number. After authentication, only the authenticated user’s information is displayed on the corresponding smartphone. Smartphone implementation is shown in Figure 2.

**Figure 3:**
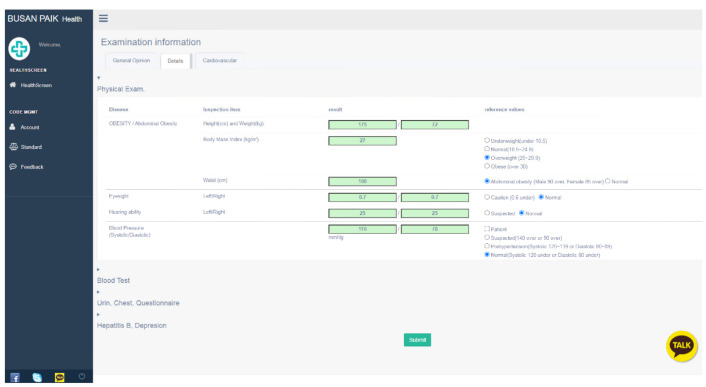
Web program to check the examination data and results in the chatbot program.

Information on study participants’ examination results was extracted from the examination results server if that server was accessible. In the case of subjects who have undergone the WGHE program at other hospitals, who would not have access to the examination results server, the examinee printed the examination results and then submitted them. The collected examination results were then stored on a different server. A separate database was built, and after the testing and service period, it was discarded to protect personal information. To test the business logic and accuracy of the data display, a separate web program was developed (Figure 3) to enter, add, modify, and delete data.

### 2.3. Program Content Development

The target diseases of the WGHE in Korea are hypertension, obesity, anaemia, diabetes, dyslipidaemia, liver disease, kidney disease, pulmonary tuberculosis, other chest diseases, and cardiocerebrovascular disease. The history-taking and physical examinations cover past personal and family history and lifestyle factors, body measurements, chest radiography, blood tests, urine tests and risk assessment for cardiocerebrovascular disease. 

The content of the program comprises two stages. The first step explains the examination results according to the test items (for example, blood pressure management state: pre-hypertensive stage, which is a blood pressure higher than normal—this is highly likely to develop into hypertension in the future) and basic recommended follow-up management (e.g., “periodic blood pressure measurement is necessary—you should eat a low-salt diet, lose weight through regular exercise, and stop smoking”). The second step requires researching and organising more detailed information than that presented in the basic results (e.g., definition and classification of hypertension) and more detailed follow-up methods than those provided as basic follow-up management (e.g., a method for self-measurement of blood pressure).

This information is provided by government agencies (National Health Insurance Service in Korea, Korea Disease Control and Prevention Agency (KDCA), National Cancer Control Institute in Korea), professional medical associations in Korea (Korean Society for the Study of Obesity, Korean Society of Hypertension, Korean Diabetes Association, The Korean Society of Lipid and Atherosclerosis, Korean Society of Nephrology, Korean Association for the Study of the Liver), and homepages of universities or general hospitals (Samsung Seoul Hospital, Seoul National University Hospital). Five occupational and environmental medicine specialists reviewed the summary of examination results, details of follow-up management, and specific information for each disease.

### 2.4. Effectiveness Survey and Applicability Interview

An eight-question survey addressing satisfaction with the chatbot was administered, including its necessity and convenience, to 23 medical personnel working at hospitals in Korea (Appendix A). They had data for the 2018 WGHE and agreed to use the chatbot program for follow-up management. 

Six nurses were interviewed, in charge of follow-up management of WGHE (Workers’ general health examination) regarding the possibility of using the chatbot program for follow-up management at the agency of occupational health management. 

They reviewed all the contents of the chatbot program for follow-up management and applied the chatbot program through an app before they took part in the interview. The interview had three aims: (1) the reason why follow-up management was difficult; (2) to evaluate the chatbot program for follow-up management; and (3) to assess the possibility of using the chatbot program in the occupational health service. 

### 2.5. A Data Analysis

The questionnaire was constructed using a 5-point Likert scale, but in the results, responses were aggregated into three categories. Very high and slightly high were classified as high, middle as moderate, and slightly low and low as low, and the results in the applicability interview were decided through a meeting of the researchers. Similar opinions were organized into one content and independent opinions were described as they were in the interview.

## 3. Results

### 3.1. Chatbot Program Contents

The program content is divided into two parts. The first includes the examination results, reference value for each test, a basic explanation of the result and actions to be taken. The second part contains detailed information about the examination results (definition and complications of target disease) and recommended managements (exercise, diet). Detailed information and sources are shown in Table 1.

### 3.2. Effectiveness Survey

Of the 30 medical personnel who agreed to use the chatbot program for follow-up management, 23 were selected as participants. Four who reported normal results (A) on all examinations (and thus did not require follow-up) and three who did not complete the survey were excluded. There were 14 males and nine females; the largest proportion (39.12%) were in their 30s. In terms of content comprehension and content specificity, 17 (73.91%) and 18 (78.26%) participants were satisfied (very satisfied, slightly satisfied), respectively. However, in terms of ease of use and ease of completion, 11 (47.83%) and 13 (56.52%) respondents, respectively, were satisfied (very satisfied, slightly satisfied), respectively, reflecting less satisfaction with the program’s ease than with content comprehension and specificity. In terms of the need for follow-up management chatbots, 21 (91.30%) respondents indicated that chatbots are needed (very necessary, slightly needed); fewer respondents (16; 69.56%) said that such chatbots could contribute to healthcare (very, very slightly). Finally, 14 respondents (60.87%) reported overall satisfaction (very satisfied, slightly satisfied) (Table 2).

### 3.3. Applicability to the Occupational Health Service

Nurses working at the agency of occupational health management highly appreciated the chatbot program, as it was especially useful as a tool for solving accessibility among the difficulties in follow-up management and as an aid for explaining examination results and recommended actions. Furthermore, they suggested that accessibility and applicability would improve if question-and-answer functions were added. However, they expressed concern about the chatbot’s inconvenience for use and accessibility for elderly workers. A summary of the interview results is shown in Table 3.

## 4. Discussion

This pilot study aimed to develop an early version of a chatbot program for follow-up management after WGHE. Another purpose was to investigate the effectiveness of use among healthcare providers and to check applicability in occupational health services among nurses in the agency of occupational health management.

The developed chatbot program is a system that generates information of follow-up management appropriate to the participants’ condition by matching the participants’ collected information (history, weight, lifestyle) with the results of the health examination. It then checks the contents of the participants’ interest from the generated information. Such a system has the advantage of first showing the overall information of follow-up management necessary for the individual’s situation. Then, from among the data provided, the participant can search more intensively for additional information of interest. Follow-up management in a health examination is not just about the individual’s interests; rather, it is an additional intervention that includes steps such as diagnosis confirmation, education, and counselling for those who need further action as a result of screening. Thus, the first step of health examination should be to provide all of the follow-up management information required by the individual.

The results of our user survey regarding the chatbot program for follow-up management showed that medical practitioners were generally satisfied in terms of content comprehension (17; 73.91%) and content specificity (18; 78.26%). About 50% of the information comprised videos or animations less than 4 minutes in length that have been provided to the general public by professional societies affiliated with the Korean Academy of Medical Science; thus, their source may be one reason for the high level of satisfaction with content and specificity due to their evidence based evidence level. Infographics, which combine information and graphics, have attracted attention as a method for effectively delivering large amounts of data and complex information [21]. Infographics help to clarify information and data or to quickly integrate difficult information by rendering it visible. The value of infographics has been demonstrated previously. One study found that healthcare professionals already use infographics to communicate medical information to their patients and public health messages to the general public [22]. Another study found that infographics proved effective when providing information to subjects who could not access, understand, and reflect on their health-management behaviour [23]. 

In terms of ease of use and ease of construction, 11 (47.83%) and 13 (56.52%) participants, respectively, were generally satisfied, reflecting less satisfaction than with content comprehension and specificity. Previous studies mentioned that ease of use positively affects the intention to use smartphone healthcare applications [24], and the higher the ease of use, the higher the intention to continue to use it [25]. Given these results, it is necessary to intensively study how to increase usability in the future.

Regarding the need for follow-up management using chatbot programs, 21 (91.30%) participants said that such chatbots were generally needed, whereas a lower proportion (16; 69.56%) believed that they could contribute to healthcare (very, very slightly). This result is similar to previous research findings showing that, although respondents were interested in healthcare applications, they did not expect them to have any effect [26]. However, the level of satisfaction in the present study was much higher than that (33.1%) for smartphone healthcare applications in a previous study [22]. This discrepancy may reflect greater satisfaction with a program that provides personalised medical information about one’s health status than with one that provides general healthcare information. Furthermore, the proportion of subjects in their 20s and 30s was high (15; 65.21%) in the present study, similar to a previous study that showed high acceptance of smartphone healthcare applications at younger ages [27]. 

Interviews with nurses working in the agency of occupational health management suggested that the chatbot would improve patient access. This improvement is because information on follow-up management could be checked at any time on a smartphone. Furthermore, they highlighted the chatbot program’s usefulness for explaining necessary follow-up management information and its value for reducing the time needed for follow-up management. Moreover, considering a previous study showing that most occupational health management agencies have difficulty developing the necessary textbooks for safety and health education in Korea [28], this chatbot program will likely be a useful tool in safety and health education for individuals and groups. The Workers Health Center, which provides follow-up management for WGHE for small-sized enterprises formed by government, previously tried to solve the accessibility; for workers to visit the Workers Health Center and for healthcare providers to visit workplaces. The chatbot program for follow-up management presented here would provide a new and simpler way to solve the problem of accessibility, which is important in the occupational health service.

This study has some limitations. First, it was a pilot study using a preliminary version of the chatbot program. The initial version of the chatbot program developed in this study provided appropriate information when a participant pressed a button. However, recent years have seen attempts to replace text input with speech recognition technology, even aiming to grasp the intention of a question using natural language processing techniques. In the next step, technologies that can facilitate a question-and-answer process will be applied. Second, a satisfaction assessment for the general public was not performed, although there are clear differences between medical providers and members of the general public from the point of view that you are already familiar with the terms of WGHE and the content that the chatbot will provide in response. However, it was necessary to test the program after creating the initial version of the chatbot program. Because it is a follow-up management program based on one’s results of a health examination, medical providers would be little significant difference to the general public at the point. In the next step, it should be planned to increase the completeness of the chatbot program and conduct satisfaction assessments among the general public. Third, the sample size is too small while proceeding with the pilot study. Likewise, no statistical analysis was performed. So, we cannot confirm statistically meaningful results. We plan to conduct large-scale research in the future to confirm statistical significance. Fourth, the chatbot program developed here focuses on comprehensive and detailed explanations of the information necessary for follow-up management. Follow-up management takes effect only when there is a change in behaviour, such as measuring blood pressure periodically or continuously exercising. However, this chatbot program cannot directly manage or support lifestyle improvement efforts. Recently, an app that helps manage smoking [19] or obesity [29] has been developed and tested. To integrate this function would be helpful for workers to change their health-related behaviour in relation to the follow-up management program. 

## 5. Conclusions 

In summary, a follow-up management program using chatbots in WGHE was developed for the first time in Korea. Chatbot users were generally satisfied, and it was confirmed that there are positive aspects in applying chatbot programs to occupational health services. For more practical follow-up management, in the future, it is necessary to develop an upgraded version of the chatbot program that includes a personalized question-and-answer process that supports behaviour changes with a large number of the general public.

## Figures and Tables

**Table 1:** Specific information and sources for follow-up management according to target diseases.

Target ds	Specific Information	Source of Information
CCVD	1. Definition of cardiocerebrovascular ds and preventive lifestyle	https://www.youtube.com/watch?v=HcM3pVhDLFY (accessed on 3 May 2019)
	2. Prevention of cardiocerebrovascular disease	https://www.youtube.com/watch?v=vttGkiDOQwc (accessed on 7 May 2019)
	3. Early symptom of cardiocerebrovascular disease	http://www.stroke.or.kr/study/movie.php (accessed on 8 May 2019)
obesity	1. Effects of exercise in obesity	http://general.kosso.or.kr/html/?pmode=exerciseObesity (accessed on 15 May 2019)
	2. Various exercise methods	http://general.kosso.or.kr/html/?pmode=movie4 (accessed on 15 May 2019)
	3. Dietary tips for weight control	http://general.kosso.or.kr/html/?pmode=nutritionTip (accessed on 15 May 2019)
	4. Various managements of obesity (meal, exercise, treatment)	http://general.kosso.or.kr/html/?pmode=obesityCure# (accessed on 17 May 2019)
	5. Eating habits of obesity by meal type	http://www.samsunghospital.com/home/healthInfo/content/contenView.do?CONT_SRC_ID=32373&CONT_SRC=HOMEPAGE&CONT_ID=4253&CONT_CLS_CD=001021005002 (accessed 17 May 2019)
	6. Physical activity/nutrition management for obesity	https://www.youtube.com/watch?v=ltuIoi53nYw (accessed on 17 May 2019)
drinking	1. Moderate drinking and proper drinking method	https://www.youtube.com/watch?v=1U58J7Hy8Qg (accessed on 18 May 2019)
	2. Self-diagnosis of alcohol dependence	http://www.nosmokeguide.go.kr/lay2/program/S1T50C55/nosmoke/noSmoke_selftest/noSmoke_selftest1_2q.do (accessed on 18 May 2019).
hypertension	1. Definition of hypertension	https://www.youtube.com/watch?v=SL_kKGCyfdo(accessed on 2 June 2019)
	2. Complications of hypertension	https://www.youtube.com/watch?v=BFEPoWNln-8 (accessed on 2 June 2019)
	3. Treatment(drug) of hypertension	https://www.youtube.com/watch?v=Kg_ds77-Y64 (accessed on 2 June 2019)
	4. Lifestyle management for hypertensive patients	https://www.youtube.com/watch?time_continue=144&v=uPETHTb69E8 (accessed on 7 June 2019)
	5. Exercise method for blood pressure control	https://www.youtube.com/watch?v=jwv62FFnBRs (accessed on 7 June 2019)
	6. Diet to control blood pressure	https://www.youtube.com/watch?v=uvrPNIimWW4 (accessed on 7 June 2019)
smoking	1. Risk of smoking, smoking cessation effect, drug therapy	https://www.youtube.com/watch?v=8hpAmvjHpuk (accessed on 8 June 2019)
	2. Self-diagnosis of nicotine addiction	http://www.nosmokeguide.go.kr/lay2/program/S1T50C9/nosmoke/noSmoke_selftest/noSmoke_selftest1_1q.do (accessed on 8 June 2019).
	3. Find a smoking cessation clinic(public health)	http://www.nosmokeguide.go.kr/lay2/program/S1T68C107/nosmoke/centermap/bogun_list.do (accessed on 8 June 2019).
Diabetes	1. Diabetes definition and classification	https://www.youtube.com/watch?time_continue=1&v=PyiZ_nPoFWo (accessed on 6 July 2019)
	2. Complications of diabetes	
	(1) diabetic foot	https://www.youtube.com/watch?v=Mhxc196ivUc (accessed on 6 July 2019)
	(2) Peripheral neuropathy	https://www.youtube.com/watch?v=Idr-BRKRlH4 (accessed on 8 July 2019)
	(3) Kidney complications	https://www.youtube.com/watch?time_continue=197&v=PN-afvhKEhI (accessed on 8 July 2019)
	3. Diabetes medication	https://www.youtube.com/watch?v=KGYgHmqBIM0 (accessed on 8 July 2019)
	4. Hypoglycemia symptoms and managements	http://www.diabetes.or.kr/general/counsel/faq_list.php?category=K (accessed on 10 July 2019)
	5. Exercise in Diabetes	https://www.youtube.com/watch?time_continue=122&v=D2A0ZudD6Vo (accessed on 10 July 2019)
	6. Diabetes Meal	http://www.diabetes.or.kr/general/food/index.php (accessed on 10 July 2019)
Dyslipidaemia	1. Types and meaning of cholesterol	http://www.lipid.or.kr/bbs/index.html?code=animation&category=1&gubun=&keyfield=&key=&mode=view&number=933 (accessed on 17 July 2019)
	2. Complications of dyslipidaemia	http://www.lipid.or.kr/bbs/index.html?code=animation&category=1&gubun=&keyfield=&key=&mode=view&number=931 (accessed on 17 July 2019)
	3. Risk factors and treatment goals of dyslipidaemia	http://www.lipid.or.kr/bbs/index.html?code=animation&category=1&gubun=&keyfield=&key=&mode=view&number=929 (accessed on 19 July 2019)
	4. Correcting lifestyle habits of dyslipidaemia	http://www.lipid.or.kr/bbs/index.html?code=animation&category=1&gubun=&keyfield=&key=&mode=view&number=930 (accessed on 19 July 2019)
	5. Exercise of dyslipidaemia	http://www.lipid.or.kr/bbs/index.html?code=animation&category=1&gubun=&keyfield=&key=&mode=view&number=926 (accessed on 19 July 2019)
	6. Diet for dyslipidaemia	http://www.lipid.or.kr/bbs/index.html?code=animation&category=1&gubun=&keyfield=&key=&mode=view&number=924 (accessed on 20 July 2019)
Anaemia	1. Comment on anaemia test results	https://youtu.be/aqxwwTln4jc (accessed on 23 July 2019)
	2. Causes, symptoms, and complications of anaemia	https://health.cdc.go.kr/healthinfo/biz/health/gnrlzHealthInfo/gnrlzHealthInfo/gnrlzHealthInfoView.do (accessed on 23 July 2019)
	3. Anaemia and Meal Therapy	http://www.samsunghospital.com/home/healthInfo/content/contenView.do?CONT_SRC_ID=09a4727a80018c1c&CONT_SRC=CMS&CONT_ID=2318&CONT_CLS_CD=001020002 (accessed on 23 July 2019)
	4. Anaemia medication	https://www.youtube.com/watch?v=qvvxdKn4y3U (accessed on 23 July 2019)
Kidney disease	1. The meaning of urine protein test	https://www.youtube.com/watch?v=t5KJk0v9ZZM (accessed on 21 August 2019)
	2. Serum creatinine and Implications of the GFR (Glomerular Filtration Rate)	https://www.youtube.com/watch?v=967CeHgF3rc (accessed on 21 August 2019)
	3. Acute kidney disease	http://www.ksn.or.kr/sub10/sub01_03.html (accessed on 23 August 2019)
	4. Chronic kidney disease	http://www.ksn.or.kr/sub10/sub01_02.html (accessed on 23 August 2019)
	5. Diet management in kidney disease	https://www.dietitian.or.kr/work/business/kb_c_kidney_glomerulonephritis.do (accessed on 23 August 2019)
Liver disease	1. Means of liver function test	https://www.youtube.com/watch?v=mmOEl4z-G9I (accessed on 27 August 2019)
	3. Possible symptoms of liver damage	https://terms.naver.com/entry.nhn?docId=2704268&cid=55588&categoryId=55588&expCategoryId=55588 (accessed on 27 August 2019)
	4. Definition and management of viral hepatitis	https://terms.naver.com/entry.nhn?docId=2704287&cid=55588&categoryId=55588 (accessed on 28 August 2019)
	5. Alcoholic liver disease management	https://terms.naver.com/entry.nhn?docId=2704307&cid=55588&categoryId=55588&expCategoryId=55588 (accessed on 28 August 2019)
	6. Standard of proper drinking amount	https://terms.naver.com/entry.nhn?docId=2704308&cid=55588&categoryId=55588&expCategoryId=55588 (accessed on 28 August 2019)
	7. Liver health, exercise and weight control	https://terms.naver.com/entry.nhn?docId=2704346&cid=55588&categoryId=55588 (accessed on 29 August 2019)
	8. Diet management of liver disease	https://www.dietitian.or.kr/work/business/kb_c_liver.do (accessed on 29 August 2019)

**Table 2:** Characteristics of participants and the effectiveness of the chatbot program for follow-up management.

Characteristics		Participant (n)	Participant (%)
Gender	male	14	60.88
	female	9	39.12
Age	20–29	6	26.09
	3–39	9	39.12
	4–49	6	26.09
	5–59	2	8.70
Content understanding	High	17	73.91
	Moderate	4	17.39
	Low	2	8.70
Content specificity	High	18	78.26
	Moderate	3	13.04
	Low	2	8.70
Convenience of use	High	11	47.83
	Moderate	7	30.43
	Low	5	21.74
Configuration convenience	High	13	56.52
	Moderate	4	17.39
	Low	6	26.09
Needs of Program	High	21	91.30
	Moderate	0	0.00
	Low	2	8.70
Contribution to health	High	16	69.56
	Moderate	5	21.74
	Low	2	8.70
Overall satisfaction	High	14	60.87
	Moderate	6	26.09
	Low	3	13.04

**Table 3:** Summary of interviews on the applicability of the chatbot program to the occupational health service.

Question	Answer
Difficulties of follow-up management in occupational health service	(1) In many cases, compliance with the follow-up management is poor for personal or business reasons. (2) Because the visit cycle is long, if you do not meet once, it will take a lot of time until the opportunity comes again. (3) There is not much time to explain the details of follow-up management. (4) It is not possible to confirm whether the workers perform the necessary follow-up management.
Evaluation of the chatbot program for follow-up ma nagement	(1) Methods of follow-up management are specific and there are a lot of videos such as YouTube, so it seems to help to understand the contents. (2) There are many visual tools such as graphs, so it is good to see results. (3) Because it is complicated to use, it seems that elderly workers will have difficulty using it. (4) Accessibility will be higher if questions and answers for interests are added.
Possibility of chatbot program for follow-up management in the occupational health service	(1) It is possible that the follow-up management information can be viewed on your smartphone at any time, so that the accessibility of the follow-up management can be improved. (2) It can be used as a material to explain the follow-up management contents to the target workers. (3) It can shorten the management time of workers in follow-up management.

## Data Availability

Data sharing is not applicable.

## Appendix A

Please mark (✓) the appropriate place for the following question after using the chatbot follow-up program				
1. Did you understand the contents explained in the chatbot follow-up program?				
① excellent	② good	③ average	④ bad	⑤ terrible
2. Was the contents of the chatbot follow-up management program specific so that it could be used realistically?				
① excellent	② good	③ average	④ bad	⑤ terrible
3. Was the chatbot follow-up program convenient to use?				
① excellent	② good	③ average	④ bad	⑤ terrible
4. Was the composition of the overall screen and buttons arranged in an easy to understand way?				
① excellent	② good	③ average	④ bad	⑤ terrible
5. Do you think it is necessary to receive specific results of your health examination through an app: such as a chatbot follow-up program?				
① excellent	② good	③ average	④ bad	⑤ terrible
6. Do you think the chatbot follow-up program can contribute to personal health promotion?				
① excellent	② good	③ average	④ bad	⑤ terrible
7. What is your overall satisfaction level when using the chatbot follow-up program?				
① excellent	② good	③ average	④ bad	⑤ terrible
8. Please give your opinion if there is a part that needs improvement in the chatbot follow-up program				
